# UltiMatch-NL: A Web Service Matchmaker Based on Multiple Semantic Filters

**DOI:** 10.1371/journal.pone.0104735

**Published:** 2014-08-26

**Authors:** Keyvan Mohebbi, Suhaimi Ibrahim, Mazdak Zamani, Mojtaba Khezrian

**Affiliations:** 1 Department of Computer, Islamic Azad University, Mobarakeh Branch, Esfahan, Iran; 2 Faculty of Computing, Universiti Teknologi Malaysia (UTM), Johor, Malaysia; 3 Advanced Informatics School (AIS), Universiti Teknologi Malaysia (UTM), Kuala Lumpur, Malaysia; Rutgers University, United States of America

## Abstract

In this paper, a Semantic Web service matchmaker called UltiMatch-NL is presented. UltiMatch-NL applies two filters namely Signature-based and Description-based on different abstraction levels of a service profile to achieve more accurate results. More specifically, the proposed filters rely on semantic knowledge to extract the similarity between a given pair of service descriptions. Thus it is a further step towards fully automated Web service discovery via making this process more semantic-aware. In addition, a new technique is proposed to weight and combine the results of different filters of UltiMatch-NL, automatically. Moreover, an innovative approach is introduced to predict the relevance of requests and Web services and eliminate the need for setting a threshold value of similarity. In order to evaluate UltiMatch-NL, the repository of OWLS-TC is used. The performance evaluation based on standard measures from the information retrieval field shows that semantic matching of OWL-S services can be significantly improved by incorporating designed matching filters.

## Introduction

The advantages of loosely coupled modeling offered by Service Oriented Architecture (SOA) have made it a superior candidate to serve as a basis for the modern enterprise systems. The services specified using the established standards of SOA are termed as Web services. According to the World Wide Web Consortium (W3C), “A Web service is a software system identified by a URI, whose public interfaces and bindings are defined and described using XML. Its definition can be discovered by other software systems. These systems may then interact with the Web service in a manner prescribed by its definition, using XML based messages conveyed by Internet protocols” [1]. Recent research works in the area of Web services focus on various issues arise throughout their life cycle. These include how to specify, discover, select, mediate, compose, invoke, and monitor Web services.

A Semantic Web service is essentially a Web service that its functionality is described using semantic annotations over an ontology. Adding semantic annotations to Web services makes them machine-understandable and intelligent. This will ease the way to automate service usage tasks. Accordingly, Semantic Web service discovery attempts to make the process of finding Web services run automatically. During such process that is often called matchmaking, the formalized description of a service request and that of a service advertisement need to be compared with each other in order to recognize common elements in these descriptions.

Current Semantic Web service discovery approaches are mainly classified into *Logic-based*, *Non-logic-based*, and *Hybrid* categories. While Logic-based approaches rely on logic inferences for the matchmaking, Non-logic-based matchmakers exploit semantics that are implicit in patterns or relative frequencies of terms in service descriptions. Hybrid approaches combine techniques from both of the previous matchmakers [2], [3].

One of the main challenges of Web service discovery is improving the performance by avoiding false results which can be either false positives (i.e., Web services in the answer set that are not relevant to the request) or false negatives (i.e., Web services that are not included in the answer set, but are relevant to the request). False positive and false negative results are respectively used to calculate the *precision* and *recall* measures of a Web service discovery approach. For any information retrieval (IR)-based approach including Web service discovery system, precision is a notion of correctness which is defined as the proportion of relevant documents retrieved by the retrieval algorithm to all of the retrieved documents, whereas recall is a notion of completeness of the approach which is defined as the proportion of relevant documents that have been retrieved to all of the relevant documents [4].

The aforementioned categories employ different strategies to perform Semantic Web service discovery and improve the performance of this process in terms of precision and recall measures. In particular, the Non-logic-based approaches aim to achieve this goal by relying on such techniques as graph matching, linguistics, data mining, or information retrieval [5]. They do not perform logical reasoning to determine the degree of similarity between two service descriptions.

In this paper, a Non-logic-based semantic matchmaker is proposed, called UltiMatch-NL. It applies two different filters to achieve more accurate results in matching requests and Web services. The proposed filters are fully semantic-based and consider various elements of a service description. In addition, a new approach is presented to weight the results of these filters and determine an overall similarity.

The Non-logic-based approaches to Web service discovery receive a request as input and return as output a list of Web services ordered by their similarity to the request. Usually, the similarity is a value between 0 and 1. Determining a threshold for the similarity value is a challenge. However, the current approaches share a common weakness, as they disregard such challenge. This study proposes the use of classification methods to eliminate the need for setting such threshold manually. The classification methods used in this study are *logistic regression* and *discriminant analysis*. These methods provide the same functionality, but follow different approaches. These classification methods are also adapted to predict the relevance of requests and Web services. There are various frameworks to describe Semantic Web services [6]. UltiMatch-NL focuses on semantic services that are described in OWL-S [7].

The remainder of this paper is structured as follows. The next section summarizes the study of related works. After that the design and implementation of UltiMatch-NL is explained. This section describes the designed filters and the technique to weight and combine their results. Then, UltiMatch-NL is evaluated and the results are analyzed. The last section concludes this paper.

## Related Works

One of the most recent Non-logic-based discovery approaches has been introduced by Plebani and Pernici [8]. They present an approach for Web service retrieval called URBE (UDDI Registry by Example). Their algorithm can evaluate the degree of similarity between a pair of Web services by comparing the related WSDL descriptions. This algorithm considers the relations between the primary constructing elements of a WSDL document and, if available, the annotations included in a SAWSDL file to improve performance of semantic matching. Li et al. [9] present an ontology-based Semantic Web services discovery framework based on WSMO. They define a semantic similarity between two terms based on a graph theory. iRDQL [10] is another Non-logic-based service matchmaker that has been proposed for OWL-S annotations.

Hybrid matchmakers also make use of the Non-logic-based matching. One of the most prominent Hybrid matchmakers for OWL-S services is OWLS-MX [11] which is inspired by another hybrid matchmaker LARKS [12]. OWLS-MX incorporates approximated IR-based matching with logical reasoning for Web services in OWL-S. In some cases, the failure results of the Logic-based matching are tolerated, resulting in more returned services. The selected token-based similarity measures used to build the OWLS-MX variants are: *cosine*, *loss-of-information*, *extended Jacquard*, and *Jensen-Shannon*. iMatcher2 [13] determines the degree of semantic matching between two OWL-S service profiles with logical reasoning and several edit- or token-based text similarity measures. Selected text similarity metrics used in this matchmaker are *Bi-Gram*, *Monge-Elkan*, *Levenshtein*, and *Jaro*. Both OWLS-MX and iMatcher2 transform structured service profile descriptions into a vector of weighted keywords to calculate the DoM between OWL-S service profiles using the mentioned similarity measures [11].

As a Hybrid matchmaker for WSMO services, WSMO-MX [14] similarly applies various filters (including Logic-based and text similarity matching) to discover and rank the relative services. This matchmaker transforms the description of *derivatives* into a weighted keyword vector and applies one of the following similarity measures: *cosine*, *extended Jaccard*, *loss-of-information*, or *weighted loss-of-information*. In SAWSDL-MX [15], the weighted keyword vectors of inputs and outputs are generated for every operation. The implemented similarity measures are the same as those considered for OWLS-MX.

The majority of current non-logical approaches are based on a Vector Space Model (VSM). VSM is one of the widely used IR models in which both the query and document are represented as vectors, with a similarity measure computed between the two. Several similarity measures have been created to compare a query vector with a document vector, with the most common being the *Jaccard*, *TF/IDF* or *cosine*, *Jensen-Shannon*, and *SFS* distance similarity measures [16]. However, a VSM relies on syntactic matching, which can create limitations, such as low recall or issues caused by synonyms (different words with same meaning) and homonyms (word with same spelling but different meanings). Accordingly, the vector representations of two documents may appear similar but actually comprise different contents.

A very few number of the Non-logic-based discovery approaches rely on semantic matching. For example, FC-MATCH [17] performs a combined logic-based and text similarity-based matching of monolithic service and query concepts written in OWL-DL. For text similarity matching, FC-MATCH relies on WordNet. Both OWLS-MX3 [18] and iSeM [19] perform structural matching between the signatures of a given Web service and request relying on a selected ontology-based concept similarity measure. Here, a concept is a phenomenon that is identified in an ontology using some abstract model along with its other relevant concepts [20]. Accordingly, the conceptual similarity refers to the relatedness between a pair of semantic concepts with respect to an ontology.

Unlike most of the related works, the approach presented in this study relies on two fully semantic-based filters for a more precise matching between Web services and requests. Accordingly, it provides an innovative technique to weight and combine the results of these filters, automatically. Moreover, the presented work proposes the use classification methods to bypass the manual setting of similarity threshold and to predict the relevance of requests and Web services. Finally, the presented approach is able to match between concepts of different ontologies.

## Design and Implementation of UltiMatch-NL

**Figure 1:**
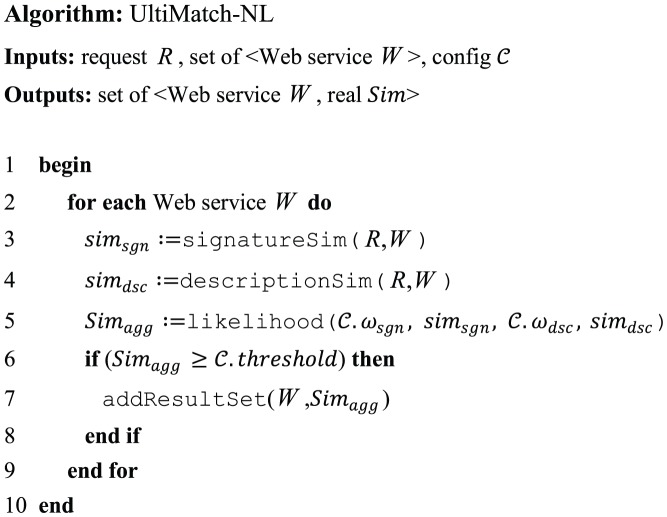
The core algorithm of UltiMatch-NL.

When determining the similarity of services, Non-logic-based approaches generally exploit techniques other than reasoning or logical expressions. The proposed Non-logic-based matchmaker UltiMatch-NL relies on various text similarity measuring techniques to calculate the matching level between a request and Web service. To achieve more accurate results, UltiMatch-NL applies two different filters called *Signature-based* and *Description-based* on the descriptions of a request and Web service. The results of these filters are then aggregated to determine their overall similarity. The core algorithm of UltiMatch-NL is depicted in Figure 1.

The inputs to this algorithm are a request, a set of Web services, and a configuration file. The configuration is comprised of the settings to customize the process of matchmaking. It may include such settings as the requested filter and the threshold of similarity, which are used by the matchmaker. The output of the algorithm includes a set of matched Web services and their similarity with the request. For a request and Web service pairing, their similarity is a real numeric value in the range of 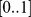. If the similarity value is equal or greater than a given threshold, the Web service and calculated similarity will be added to the result set.

Based on the principles of the Signature-based filter which is proposed in Section 3.1, the function signatureSim() calculates the similarity of the concepts included in both the inputs and outputs of a request and Web service. The function descriptionSim() implements the Description-based filter which is described later. This filter adopts a semantic similarity metric to calculate the similarity between the textual descriptions of the request and Web service.

The function likelihood() aggregates the results of Signature-based and Description-based filters to calculate the overall similarity of the request and Web service using the following formula:
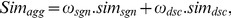
(1)where 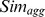 is the aggregated similarity, 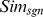 and 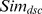 are the respective results of Signature-based and Description-based filters, and 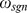 and 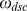 are their corresponding weights. These weights are extracted from configuration 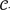. The weights determine the degrees to which different parts of a service description are regarded in the matchmaking process. The weighting of the proposed Non-logic-based filters is described in detail in Section 3.3.

### 3.1. Signature-based Filter

The signature of a Web service or request can be defined in terms of the inputs and outputs included in the description of that service. The inputs and outputs of a service are described as a set of concepts defined in some domain-specific ontology. The *Signature-based* filter determines the similarity level of a request and Web service by comparing their respective signatures.

The Signature-based filter relies on a maximum weight bipartite graph (MWBG) to obtain the more accurate similarity between the elements of two comparative sets. In the following, the principles of MWBG are introduced and adapting this graph to the proposed Signature-based filter is described.

#### 3.1.1. Matching in Maximum Weight Bipartite Graph

Suppose that 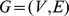 is an undirected graph, where 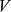 is the set of vertices and 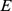 is the set of edges. This graph is termed bipartite if 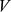 is divided into two categories and 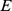 exists only between the vertices of the different categories. The matching of two sets of components can be realized using a bipartite graph approach [21]. Given a pair of sets 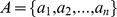 from the advertised components (e.g., inputs or outputs of the published Web services) and 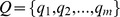 from the queried components (e.g., inputs or outputs of the requested Web services), an undirected bipartite graph can be constructed as follows:
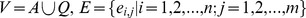
(2)If each edge 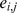 is associated with a weight, the created graph is termed a weighted bipartite graph. The weight of edge 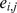 can be allocated by means of the similarity between vertices 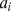 and 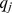.

A matching 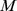 is defined as a subset of edges 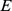 such that for all vertices 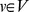, at most one edge of 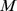 is incident on 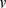. A maximum weight matching in a weighted bipartite graph is defined such that the sum of the weights of the edges in the matching is maximized. In this manner, the problem of determining the maximum matching of two sets of components can be transformed into a problem of determining the maximum weight matching of their respective weighted bipartite graph. This problem requires the calculation of an optimal pairwise assignment between the components of two sets. The well-known *Hungarian* algorithm has been proposed to solve this assignment problem [22]. In this work, an implementation of the Hungarian algorithm is used as presented in [23]. This implementation is an enhancement to the original algorithm that solves the assignment problem for a bipartite graph with two node sets of different cardinalities.

**Figure 2:**
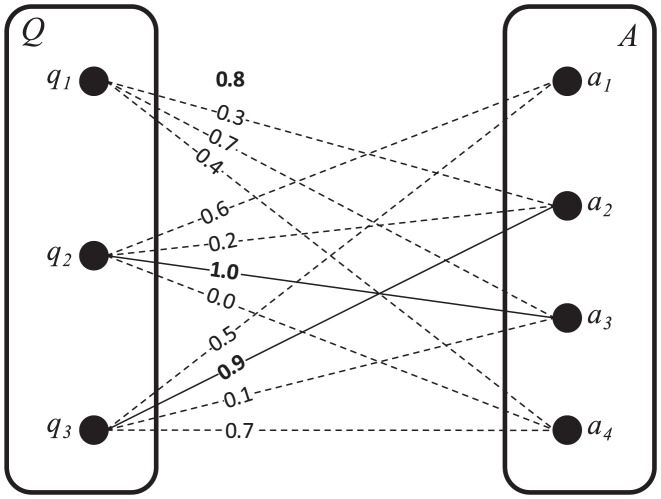
Matching components in a weighted bipartite graph.

To obtain the maximum weighted assignment between a set of advertised components 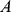 and a set of queried components 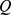, suppose that 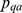 is a binary variable indicating whether 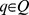 and 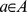 have been paired, and the weights of the edges are determined by a function 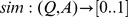. Then, function 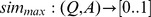 is defined as follows:
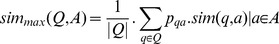
(3) This result means that the maximum similarity of the two component sets is the average of the maximum similarities found for the components in 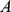 for each component in 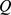. Figure 2 depicts an example of the matching components in a weighted bipartite graph.

The weights are assigned with a similarity function. In addition, components 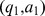, 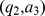, and 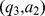 are paired due to their higher similarity in each group. Equation 3 is applied to obtain the maximum similarity between the two sets of queried components 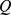 and advertised components 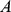. The result is as follows:
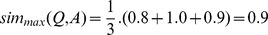



#### 3.1.2. Adapting Maximum Weight Bipartite Graph to Web Service Discovery

To compute the signature-level similarity value of a request 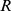 and Web service 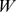, the maximization function in Equation 3 is applied separately to the sets of input and output concepts of 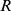 and 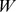. However, applying the maximization function to the input concepts is different from the output concepts as in the following.

In a perfect match, the inputs and outputs of 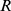 are same as those of 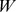. Any contravention of this rule affects their similarity and matching. In particular, any deficiency in both providing all of the inputs required by 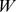 and offering all of the outputs requested by 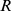 are the primary reasons for a decrease in the similarity of 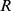 and 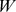. Thus, any approach that calculates the similarity of services should consider these cases.

**Figure 3:**
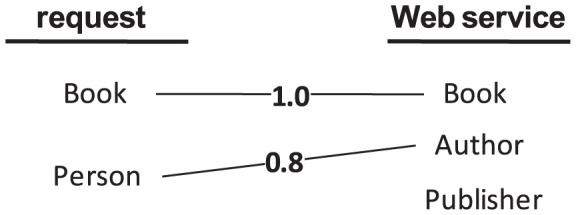
Corresponding concepts of a request and Web service.

As an example, Figure 3 depicts a request and Web service in terms of their concepts and the pairwise similarities between the corresponding concepts. For simplicity, the discarded edges are not shown here. Let these concepts be the inputs of the request and Web service. Then the request does not provide all of the inputs required by the Web service. If these concepts are assumed to be outputs, then the Web service offers all of the concepts (equal or similar) required by the request plus an additional output *publisher*.

To resolve this issue, if Equation 3 is applied on the input concepts of 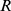 and 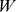, the cardinality of the set associated with 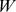 is decisive. In contrast, the cardinality of the set associated with 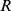 is decisive for their output concepts:
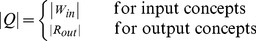
(4)This result implies that if 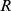 does not provide all of the inputs required by 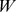 or if 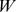 does not offer all of the outputs requested by 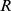, then their similarity decreases. For the example in Figure 3, applying Equations 3 and 4 results in the following similarity values:
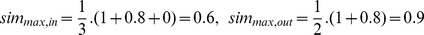



#### 3.1.3. Determining the Weights of Edges in Maximum Weight Bipartite Graph

To adapt Equation 3 to the proposed Signature-based filter, the function 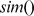 calculates the similarity between a pair of input or output concepts with respect to their relationship in an ontology. In this work, each input or output concept of either a request or Web service is assumed to arbitrarily refer to any ontology. In actuality, one cannot restrict different service descriptions to employ the same ontology, even if they are defined within the same application domain. Thus, to support the aforementioned assumption, the following scenarios are proposed to be considered:

- *Two comparative concepts use the same ontology*: In this case, the similarity is calculated with respect to their domain-specific ontology. Such an ontology is generally developed by a domain expert and includes the terms related to a specific application domain.
- *Two comparative concepts use different ontologies*: In this case, the similarity is calculated with respect to a general-purpose ontology. The WordNet ontology is used because it is a lexical database of English words that are organized according to their semantic relationships.

**Figure 4:**
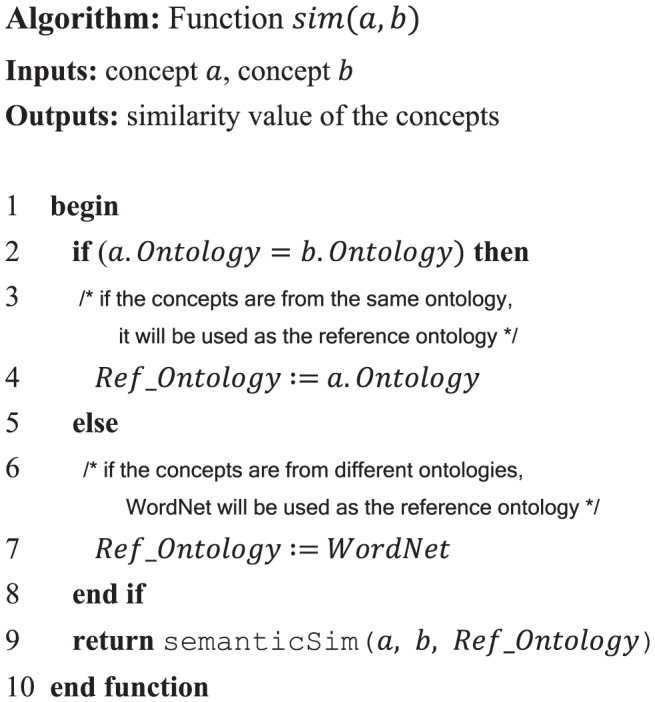
The algorithm of function *Sim*(*a,b*).

The algorithm in Figure 4 depicts the effects of these scenarios on the function 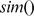 in Equation 3.

Within the algorithm, the function semanticSim() is responsible for evaluating the similarity between the input concepts with respect to their relationship in the reference ontology. Several approaches have been proposed to measure the semantic similarity and relatedness among the terms [24], [25]. Based on the evaluation of similarity measures performed in the existing literature [26], intrinsic information content-based approaches provide higher accuracy than other measures. This result has no dependency on data availability, data pre-processing, or tuning parameters for a concrete scenario. These measures are independent from domain-specific ontologies because they rely on the most commonly available features of the ontologies. They also maintain a low complexity level of computations, increasing their scalability in terms of processing large sets of data. These features are especially useful when implementing a generic approach for Semantic Web service matchmaking.

In this work, the intrinsic information content (IC)-based measures proposed by Jiang and Conrath [27] and Lin [28] are adopted. As stated in [26], these are the best performing measures for computing the semantic similarity among terms. Both of the selected measures are based on the notion of IC in information theory. The IC of the concept 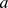 is computed by the negative logarithm of its appearance probability, 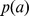, in a taxonomy.
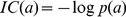
(5)The measure proposed by Jiang and Conrath is based on quantifying the length of the taxonomical links as the difference between the IC of a term and its subsumer. When comparing a pair of terms 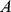 and 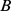, their distance is computed as follows:
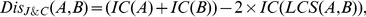
(6)where 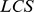 denotes the least common subsumer for a pair of terms in an ontology [27].

According to Lin [28], the similarity between a pair of terms 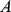 and 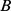 in a taxonomy can be measured as the ratio between the amount of information required to state their commonality and the information required to fully describe their status. This measure is computed as follows:
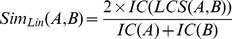
(7)Within UltiMatch-NL, the selected similarity measures will be adopted by separate variants of the Signature-based filter.

#### 3.1.4. Implementing the Signature-based Filter

**Figure 5:**
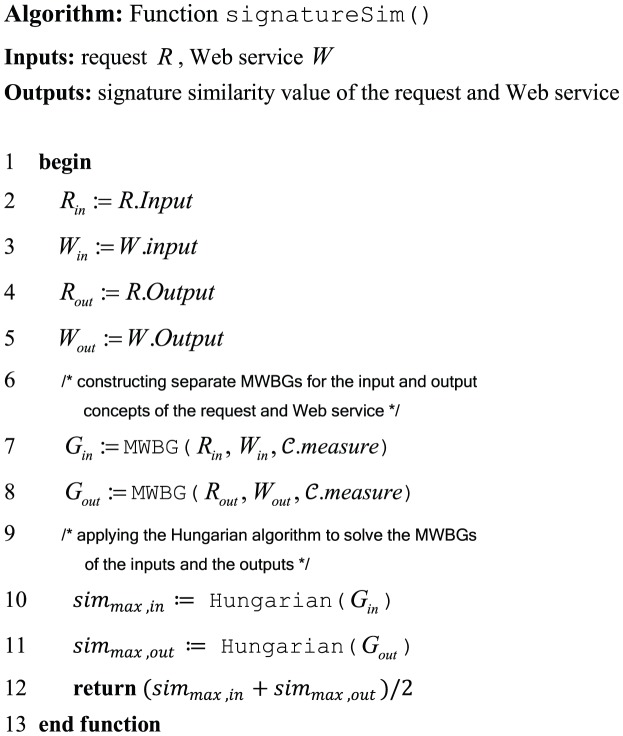
The algorithm of function *signatureSim*().

The function signatureSim() in Figure 1 implements the Signature-based filter. The algorithm of this function is depicted in Figure 5. It uses a request and a Web services as input and returns the similarity of their signatures as a real number in the range of 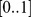.

The algorithm first extracts input and output concepts of the request and the Web service. It then constructs separate MWBGs for the extracted input and output concepts based on the principles described in Section 3.1.1. The weights of edges in MWBGs are determined using the instructions in Section 3.1.3 and the selected measure which is retrieved from configuration 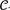. To achieve the maximum similarity values of the input and output concepts, the extended Hungarian algorithm (cf. Section 3.1.1) is applied on their MWBGs considering the adaptation principles of Section 3.1.2.

Finally, the overall similarity of the request and the Web service is defined as the average of distinct similarity values obtained for the input and output concepts of their signatures. Because it is assumed that the individual similarities of inputs and outputs have the same importance on the overall similarity they are weighted equally. However, in specific cases where inputs and outputs do not have the same priority, their individual similarities could be weighted differently.

### 3.2. Description-based Filter

In general, each Web service includes a textual description that briefly describes its functionality, thus making the code more understandable. Textual descriptions have been considered important information elements for discovery by several matchmakers [14], [11], [29]. However, the existing matchmakers generally rely on the classic vector-based similarity measures (e.g., *cosine*, *Jaccard*, *Jensen-Shannon*, and *loss of information*) to compare the similarity of the descriptions. A major disadvantage of these vector-based measures is that they consider a descriptive text syntactically as a “bag of words” and do not account for the semantics. This oversight leads to faulty similarity estimations.

For example, suppose that the following textual descriptions are used to describe a Web service and a request:

*Description of Web service: “We sell tickets for flying”*

*Description of request: “I want to travel by airplane”*

These descriptions have similar meanings but no words in common. Thus, syntactic measures are unable to detect the high level of similarity between these descriptions and wrongly return the Web service as irrelevant to the request. Conversely, the following descriptions of a Web service and a request have many common words with different meanings:

*Description of Web service: “A service to provide the statistics for the world cup in year 2014”*

*Description of request: “A service to provide the statistics for the population of the world in year 2014”*

Again, the syntactic measures detect a high level of similarity between these descriptions and wrongly return the Web service as relevant to the request.

In this section, a filter is designed to measure the similarity of services based on their textual descriptions. As this filter uses the descriptions of services, it is called *Description-based* filter. This filter considers both the syntactic and semantic information of textual descriptions when computing the similarity of a request and Web service.

#### 3.2.1. Adopting Semantic Similarity Metric in Web Service Discovery

**Figure 6:**
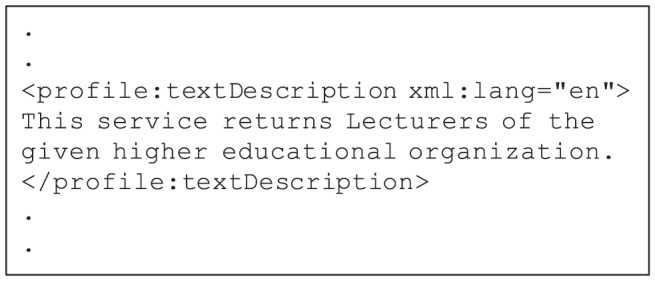
Using *textDescription* element to describe OWL-S services.

To enable the Description-based filter, the request and Web service should supply a short text written in natural language to describe their requested or provided functionality, respectively. This descriptive text is typically included in a service's non-functional property (NFP). In OWL-S, the textDescription element of the service profile is considered to include the textual description of a service as depicted in Figure 6.

**Figure 7:**
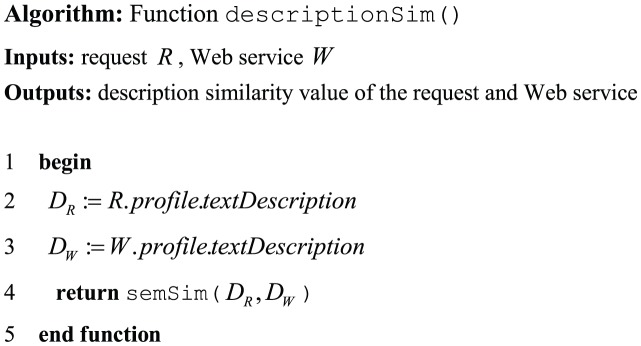
The algorithm of function *descriptionSim*().

The function descriptionSim() in Figure 1 implements the Description-based filter. The algorithm of this function is shown in Figure 7. This algorithm uses a request and a Web services as input and returns the similarity of their descriptions as a real number in the range of 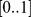. The textual descriptions are first extracted from the *textDescription* NFPs of the request and the Web services. The function semSim() then measures the semantic similarity between the extracted pair of descriptions. This function adopts the approach proposed by Pirró [30] to calculate the similarity of a pair of service descriptions. In this approach, the author has designed, implemented, and evaluated a new semantic similarity metric. The devised metric, termed *P*&*S*, combines the feature-based and information theoretic theories of similarity to achieve more accurate results.

A number of semantic similarity measuring techniques have been proposed to address the aforementioned requirements for precise evaluation [31], [30], [25], [32]. An increasing number of applications in many text-related research areas employ these techniques including text summarization, Web IR, document retrieval, text mining, paraphrase recognition, and question answering. In general, these applications obtain both semantic information from a lexical resource (e.g., WordNet) and syntactic information through a deep parsing process to capture the meanings of provided texts and measure their semantic similarity [32].

In the Description-based filter, to capture the semantic similarity between two services, the *P*&*S* metric is applied to their textual descriptions because based on the experimental evaluation in [30], this metric has outperformed other similar approaches. However, the *P*&*S* metric can be easily replaced with any similar metric of interest.

### 3.3. Proposed Technique to Weight Filters of UltiMatch-NL

To combine the Signature-based and Description-based filters, appropriate weights should be associated with their results. These filters can be weighted either manually or automatically as in the following.

- *Manual Weighting*: refers to determining the weights based on the user's preferences. In this case, the function likelihood() in Figure 1 returns the value of similarity (VoS) between a request and Web service. To obtain the standardized VoSs, the following assumptions hold on the elements of Equation 1:
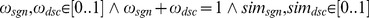
(8)
  These result in 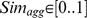.
  The manual weighting of different parts of service descriptions is subjective and imprecise because it is difficult to state that one type of information is more important than another. Furthermore, the process of determining the weights based on the manual investigation of service descriptions can be cumbersome (if not impossible) when considering a large number of services. In addition, this process should be repeated with the replacement of a Web service repository.
- *Automatic Weighting*: To overcome the shortcomings of manual weighting, an automatic weighting approach is proposed in this work. Deriving weightings for filters of UltiMatch-NL is described in detail in Section 3.3.1. Two statistical methods are applied to analyze the existing data and estimate the optimal weights. In the case of automatic weighting, the function likelihood() in Figure 1 refers to the implementation of the selected estimating method. Furthermore, 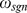 and 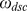 of Equation 1 represent the coefficients derived from the application of the estimation method on the training dataset. In addition the function likelihood() returns the probability of relevance (PoR) of the Web service to the request. Thus, the result (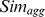) is limited to 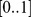.

#### 3.3.1. Deriving Weightings for Filters of UltiMatch-NL

To derive weightings for various filters of UltiMatch-NL, two statistical methods are chosen, namely, logistic regression and discriminant analysis. These methods have essentially been designed to evaluate the associations between different variables and a categorical outcome. Logistic regression and discriminant analysis can both be used to predict the probability of a specified outcome using available variables [33]. Both methods are used extensively in numerous disciplines, such as business management [34], civil and environmental issues [35], medical and health sciences [33], social sciences [36], and image processing applications [37]. In addition, the comparative evaluations of logistic regression and discriminant analysis have proved their appropriate performance in numerous experiments [38], [39], [33], [34].

In the following, the properties of these methods are summarized and their applicability for the estimation of optimal weights is examined.

#### 3.3.2. Logistic Regression Method

Logistic regression [40] is a type of regression analysis used to find the best model to describe the relationship between an outcome (dependent or response) variable and a set of predictor (covariate or explanatory) variables. In contrast to ordinary linear regression, where the dependent variable has a continuous nature, logistic regression considers categorical responses, especially binary or dichotomous variables. A binary logistic regression refers to an observed outcome with two possible types (e.g., yes versus no, male versus female, or pass versus fail).

Similar to ordinary regression, logistic regression provides a coefficient that measures the partial contribution of each of the covariates to variations in the dependent variable. Unlike ordinary regression, the user's prediction from the knowledge of covariates and coefficients is not a numerical value of a dependent variable but rather the probability that it belongs to a particular group [41]. To accomplish this goal, let the conditional probability that the outcome is present be denoted by 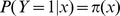. The logit function (i.e., the logistic transformation) of the logistic regression model is given by the following equation:
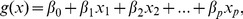
(9)where 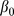 is the constant of the equation, 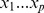 are predictor variables and 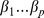 are their respective coefficients. The general method of estimation of the logit models is the maximum likelihood, where the probability of obtaining the observed results given the fitted regression coefficients is intended to be maximized. The logistic regression model is:
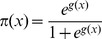
(10)Here, 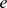 is the base of the natural logarithm [40].

#### 3.3.3. Discriminant Analysis Method

Discriminant (or discriminant function) analysis [42] is used to determine which continuous variables discriminate between two or more classes of objects or events. A fundamental assumption of this method is that each of the continuous (independent) variables is normally distributed. Discriminant analysis creates a model that aims to determine the most efficient way to distinguish between classes or groups and to maximize the possibility of classifying cases into their respective groups.

Similar to ordinary regression, discriminant analysis determines a linear model that will predict to group to which the case belongs. The analysis creates a discriminant function, which is a linear combination of the weightings and scores on these variables. The score of the discriminant function for the 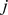
th group is calculated by the following equation:
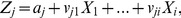
(11)where 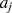 is a constant, 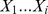 are the discriminating variables, and 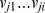 are their respective coefficients. These coefficients maximize the distance between the means of the dependent variable. In fact, the discriminant function is expected to maximize the distance between groups, i.e., create an equation that has a strong discriminatory power between them. The number of discriminant functions is the number of groups minus one. Once the discriminant functions are determined, the groups are differentiated. The utility of these functions can then be examined through their ability to correctly classify each case according to its *a priori* group. Classification functions are then derived from the linear discriminant functions to achieve this purpose [43].

#### 3.3.4. Proposed Model to Apply the Statistics-based Methods

Both the logistic regression and discriminant analysis methods can be applied to estimate the PoR of a Web service to a request. The process is outlined below.

To use the aforementioned statistics-based methods to analyze the data, one must first define a model. The model is generally a composition of dependent and independent variables. It is assumed that the weighted linear combination of similarities on various levels (the input features) predicts the membership of a Web service to a request relevance set. This model is described by the following linear equation:
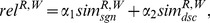
(12)where the dependent variable 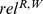 denotes the relevance of a request 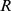 and Web service 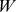, the independent variables 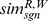 and 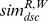 denote the similarity between 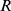 and 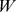 on *Signature* and *Description* levels, respectively, and the importance of these similarities is denoted by the coefficients 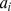. The aim is to estimate these coefficients such that this model can accurately predict their relevance for a request and Web service pairing. Prior to this estimation, the model must be trained on the existing data.

To train the model, a set of requests and Web services and their predefined relevance is required. For instance, the test collection OWLS-TC [44] used in this work provides these data. OWLS-TC contains a number of services and queries. Each query is associated with a relevance set that includes services that are defined as relevant to that query.

In all of the test collections, relevance is defined according to the standard TREC working definition of binary relevance [45], i.e., services are judged as either *relevant* or *not relevant* to the queries. To place these discrete relevance grades in the defined model, they should be mapped to the following numerical values:
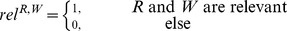
(13)Because the output of the model in Equation 12 is categorical, it is suitable to induce the aforementioned statistics-based methods to investigate the relationship between this result and the independent variables.

For the training phase, a subset of all known pairs of requests and Web services may be selected. Each selected pair of a request 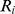 and Web service 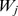 is represented by a vector as follows:
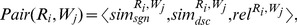
(14)in which the individual similarities of 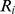 and 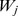, based on the different levels and their relevance, are the dimensions of the vector. For each request and Web service pairing, UltiMatch-NL links the pair and also retrieves its predefined relevance. The computed similarities and their relevance are stored in a vector. All of the vectors 
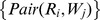
 are considered as a training set. These vectors constitute a matrix in which each pair of request and Web service yields one row with three columns. A sample matrix is depicted below.
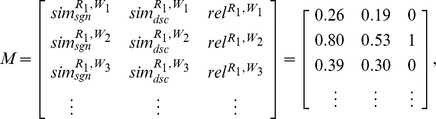
where the first three rows contain the data for the pairs of requests and Web services 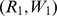, 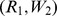, and 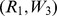. One can also observe that the first and third pairs are not relevant, whereas the second one is relevant according to the mapping shown in Equation 13.

Given the model shown in Equation 12 and the matrix containing the training set, each of the selected statistical methods may be applied to estimate the coefficients of this model. As an example, assume that the application of these methods on the previous matrix 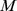 outputs the following estimates. The estimated coefficients can be used to predict the relevance of a new request and Web service pairing.

Suppose that for a request 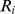 and Web service 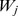, the results of the UltiMatch-NL filters are 
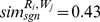
 and 
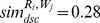
. Given these computed similarities and the estimated coefficients of Table 1, each of the aforementioned statistical methods could be applied to predict the dependent variable in Equation 12. In this case, these methods are adopted to predict if 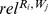 is 1. The result will be the PoR of 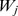 and 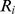. For this example, the estimated probabilities are shown in Table 2.

**Table 1:** Applying the statistics methods on sample data.

Method	Coefficient	
	*α*_1_	*α*_2_
logistic regression	12.99	11.55
discriminant analysis	15.37	15.30

**Table 2:** Estimated probabilities of applying the statistics methods on sample data.

Method	PoR of *W_j_* and *R_i_*
logistic regression	74%
discriminant analysis	65%

Both the logistic regression and discriminant analysis methods provide similar functionalities by using different approaches. To choose between these methods, they should be evaluated and compared with respect to a specific dataset. To evaluate the applied methods, the quality of their prediction will be measured. The purpose of this phase is to select the statistical method that can more accurately predict the relevance of requests and Web services. However, the result is heavily dependent on the dataset employed. Details of this evaluation are provided in the next section.

## Analysis and Evaluation

In this section, UltiMatch-NL is evaluated. Precision and recall [46] metrics as well as their derivatives are used to measure the performance of this matchmaker. In particular, the evaluation strategy of macro-averaged precision is adopted that computes the mean of the precision values for the retrieved documents of all queries in the test collection at equidistant standard recall levels. Moreover, the tool Semantic Service Matchmaker Evaluation Environment (SME2) (cf. http://projects.semwebcentral.org/projects/sme2/) is being used. SME2 is designed as an integrated environment that provides an extensible framework for the testing of different semantic matchmakers in a consistent way. Different test collections and matchmakers can be added as plug-ins to the environment to conduct performance experiments. The framework can be easily extended to support various types of matchmakers. This tool has been used in the yearly S3 contest. To make use of SME2, UltiMatch-NL is developed as a plugin for this framework.

For this evaluation, the test collection OWLS-TC (cf. http://projects.semwebcentral.org/projects/owls-tc/) is being used. The choice of OWLS-TC was motivated by its widespread use for evaluating semantic service retrieval algorithms [47]. More specifically, OWLS-TC version 2.2 revision 2 [44] has been selected. This test collection consists of 1,007 Semantic Web services written in OWL-S 1.1 (and, for backward compatibility, OWL-S 1.0) from seven application domains consisting of *education*, *medical care*, *food*, *travel*, *communications*, *economics*, and *weapons*. It also provides a set of 29 test queries. Each Web service is judged either relevant or not relevant to a query. The semantic annotations of all services are based on references to 34 OWL ontologies in total.

**Figure 8:**
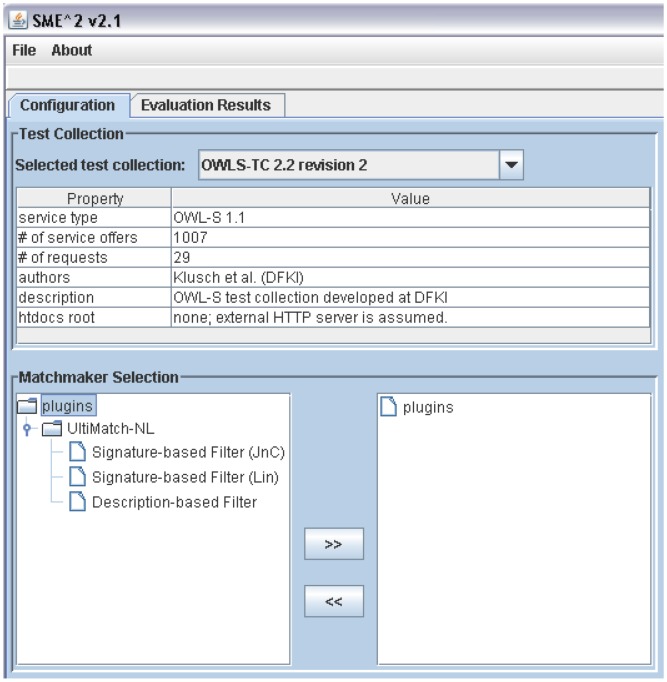
Configuring the test collection and the plugins of UltiMatch-NL.

Because UltiMatch-NL comprises Signature-based and Description-based filters, the performance of each filter is investigated separately and in combination. Figure 8 shows the configuration part of SME2 where the plugins of various filters of UltiMatch-NL are added for evaluation. In addition, OWLS-TC is considered as the test collection.

For the Signature-based filter, the sets of input and output concepts of each request and Web service are extracted. Based on the *Jiang and Conrath (J*&*C)* and *Lin* measures selected to assess the semantic similarity between the concepts, two variants of the Signature-based filter are considered for this evaluation. This filter is able to resolve the relationship between the concepts of heterogeneous ontologies. The extJWNL API (cf. http://extjwnl.sourceforge.net) is used to handle the WordNet processing.

**Figure 9:**
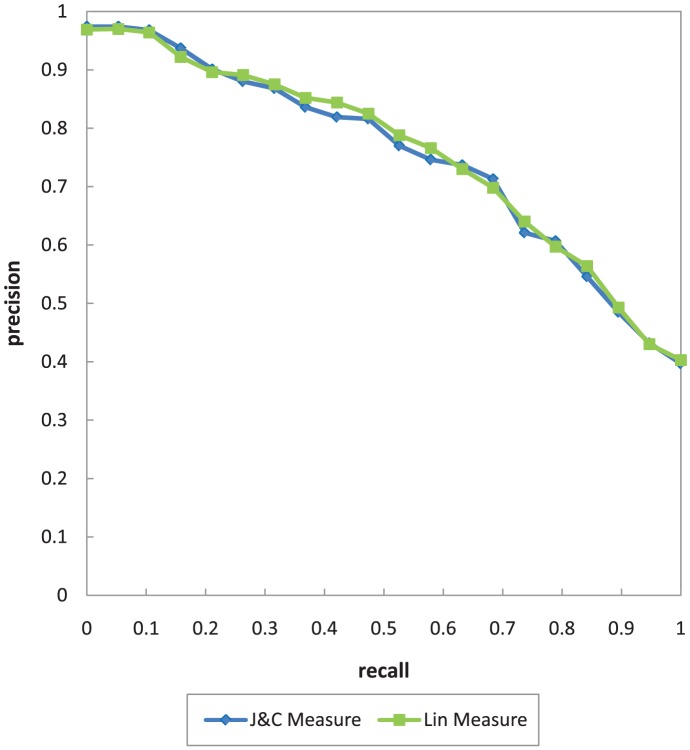
The R/P graph for various Signature-based filters of UltiMatch-NL.

In this evaluation, precision, recall, and mean average precision (MAP) metrics are selected with the strategy of macro-averaged precision over 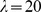 recall steps. The recall/precision (R/P) graph is depicted in Figure 9.

As shown in Figure 9, both variants of the Signature-based filter exhibit similar results, but the one relying on the Lin measure performs slightly better with respect to the current test collection. Therefore, this measure is exclusively used to configure the Signature-based filter for the rest of experiments in this work.

In the next experiment, the performance of the Description-based filter is evaluated. To apply the Description-based filter on a request and Web service pairing, their respective *textDescription* NFPs are extracted. The *P*&*S* metric is then applied to compute the semantic similarity between these descriptions. The API provided in the Java WordNet Similarity Library (cf. http://grid.deis.unical.it/similarity) is used to implement this metric.

**Figure 10:**
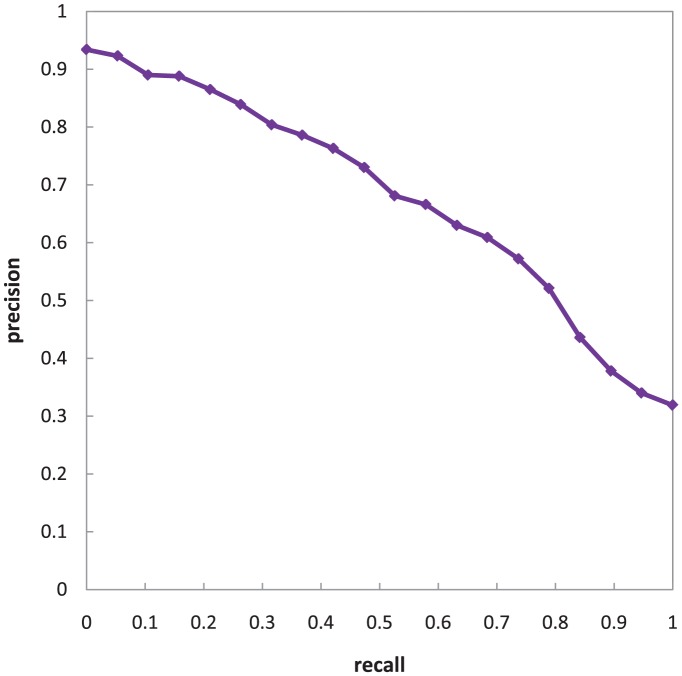
The R/P graph for Description-based filter of UltiMatch-NL.

The evaluation settings are same as those for the Signature-based filter. The results of the evaluation can be viewed in Figure 10.

To enable or disable each of the aforementioned filters, the corresponding weights assigned to the *Signature* and *Description* similarities (i.e., 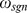 and 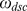 in the algorithm of Figure 1) are set to one and zero, respectively. However, to exploit both of these filters, their results should be aggregated using an appropriate weighting mechanism.

### 4.1. Evaluating the Technique to Weight Filters of UltiMatch-NL

The application of two statistical methods to weight the filters of UltiMatch-NL and to estimate the similarity of a request and Web service has been studied in Section 3.3. To select the most appropriate method, the following steps are followed. All analyses are performed offline using PASW Statistics version 17 (SPSS, Inc., Chicago, IL, USA).

The first step is to train the model proposed in Equation 12, i.e., 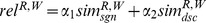 using the data of OWLS-TC. The entire sets of requests and services (including 29 requests and 1,007 Web services) are selected from this test collection. For each possible pair of request and Web service, UltiMatch-NL calculates their similarity on both *Signature* and *Description* levels and retrieves their predefined relevancy. These values are stored in a vector, as defined in Section 3.3.4. All of these vectors constitute a matrix that becomes the training set.

The dependent variable 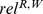 and covariates 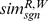 and 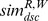 from the aforementioned model enter into both the logistic regression and discriminant analysis methods using the data from the training set matrix. The assumptions for the two methods are fulfilled. The covariates follow the multivariate normal distribution and are independent. For the discriminant analysis, group membership is mutually exclusive (i.e., a case does not belong to more than one group) and collectively exhaustive (i.e., all cases are members of a group). Moreover, according to the following tests, within-group variance-covariance matrices are equal across all groups. The practical results of applying the logistic regression are depicted in Table 3.

**Table 3:** Equation coefficients of logistic regression method.

Covariate	B	Sig.
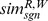	14.372	0.000
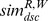	12.583	0.000

This table summarizes the effect of each predictor. For both of the predictors, the significance level of the Wald statistic is less than 0.05 (exactly zero), indicating that these parameters are useful to the model. The estimated coefficients shown in column B are used by the method to make predictions.

The discriminant analysis results are depicted in Table 4. Classification functions use coefficients in Table 4 to assign cases to groups. The canonical relation is a correlation between the discriminant scores and the levels of the dependent variable [48].

**Table 4:** Classification function coefficients of discriminant analysis method.

Covariate	*rel^R,W^*	
	0	1
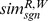	10.037	23.058
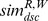	5.422	16.612

In this test, the high canonical correlation of 0.77 indicates a function that discriminates well (one is perfect). Furthermore, according to the tests of equality of group means, both of the variables in the discriminant model are significant (Sig. <0.10).

Box's *M* test tests the equality of covariance matrices across groups. Although the significance level of the test is zero, another analysis reveals that using a separate-groups covariance matrix does not significantly change the classification results (only 0.1%). Box's *M* can be overly sensitive to large data files, which is likely what occurred in this case [48].

After training the model, the next step is to evaluate the classification methods by making predictions against the test set. The same set of training data is used for the evaluation. Because this dataset already contains a relevance set for each request, it is easy to determine whether the method's predictions are correct. The logistic regression results are depicted in Table 5.

**Table 5:** Classification results of logistic regression method.

Observed		Predicted		
		*rel^R,W^*		Percentage Correct
		0	1	
*rel^R,W^*	**0**	20,489	883	95.9
	**1**	1581	6,250	79.8

For each case, the predicted response is one if that case's model-predicted probability is greater than the cutoff value; a default of 0.5 is used. In assessing all of the cases, 20,489 of 21,372 are not relevant, and 6,250 of 7,831 relevant Web services are classified correctly. Overall, 91.6% of the cases are classified correctly.

Table 6 presents the classification results obtained using discriminant analysis. In assessing all of the cases, 20,429 of the 21,372 are not relevant and 6,369 of 7,831 relevant Web services are classified correctly. Overall, 91.8% of the cases are classified correctly.

**Table 6:** Classification results of discriminant analysis method.

Observed		*rel^R,W^*	Predicted Group Membership		Total
			0	1	
**Original**	**Count**	**0**	20,429	943	21,372
		**1**	1,462	6,369	7831
	**%**	**0**	95.6	4.4	100.0
		**1**	18.7	81.3	100.0

Apart from the above results, another approach is adopted to independently evaluate the performance of the aforementioned methods. The Receiver Operating Characteristics (ROC) graph is a useful way to evaluate the performance of the classifiers that categorize cases into one of two groups [49]. A ROC graph demonstrates the accuracy of a classifier in terms of two measures: *specificity* and *sensitivity*. *Specificity* is the probability that a negative case (i.e., a not relevant Web service) is correctly classified. The false positive rate is identified as *1 – specificity* and is plotted on the *X* axis of a ROC graph. *Sensitivity* is the likelihood that a positive case (i.e., a relevant Web service) is correctly classified. *Sensitivity* identifies the true positive rate and is plotted on the *Y* axis of a ROC graph. By plotting the ROC graphs for various classifiers on the same axes, one can determine which approach performs better. The evaluation process is outlined below.

**Figure 11:**
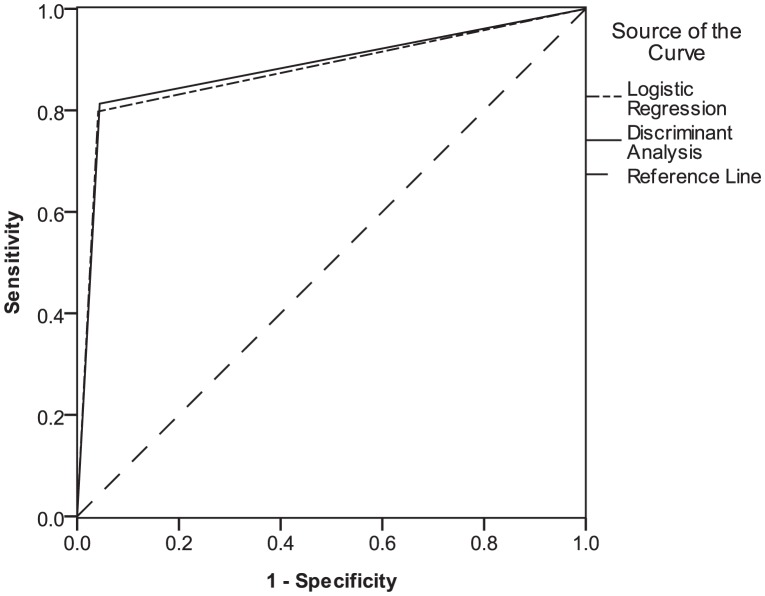
ROC graph for logistic regression and discriminant analysis methods.

For every request and Web service pairing, the calculated similarity values on various levels and the learned coefficients from the application of each of the statistical approaches in the training phase enter the model proposed in Equation 12, i.e., 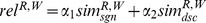. Logistic regression and discriminant analysis are then separately used to predict the relevancy of that request and Web service. After processing all pairs of requests and Web services, the ROC graph is plotted for each method using the predicted and actual retrieved relevancies. A single point is produced for each method corresponding to its overall false positive and true positive rates. The ROC graph is illustrated in Figure 11.

The point (0,1) is the perfect classifier, which classifies all positive cases and negative cases correctly. The false positive rate of this point is zero (none), and the true positive rate is one (all). The point (0,0) represents a classifier that predicts all cases to be negative, whereas the point (1,1) corresponds to a classifier that predicts every case to be positive. Point (1,0) is the classifier that is incorrect for all classifications. In a ROC graph, the AUC can be used as a measure of accuracy [49]. As shown in Figure 10, the ROC curves of the aforementioned methods indicate that the discriminant analysis and logistic regression have similar levels of accuracy (88.5% versus 87.8%).

In summation, both logistic regression and discriminant analysis are applied to predict the relevancy of Web services and requests based on their similarities at individual levels. In addition, the observed values of the outcome are compared with the predicted values to determine which method more accurately predicts the relevancies. Both statistics converged to similar results. Their overall classification rate was good, and either method can be used to predict the relevancy of a Web service and request. Although discriminant analysis slightly exceeds logistic regression in terms of classification correctness, the differences in the AUCs were negligible, indicating no discriminating difference between these methods. However, discriminant analysis is being chosen along with its learned coefficients to integrate the results of the UltiMatch-NL filters in the following experiment.

### 4.2. Evaluating UltiMatch-NL

**Figure 12:**
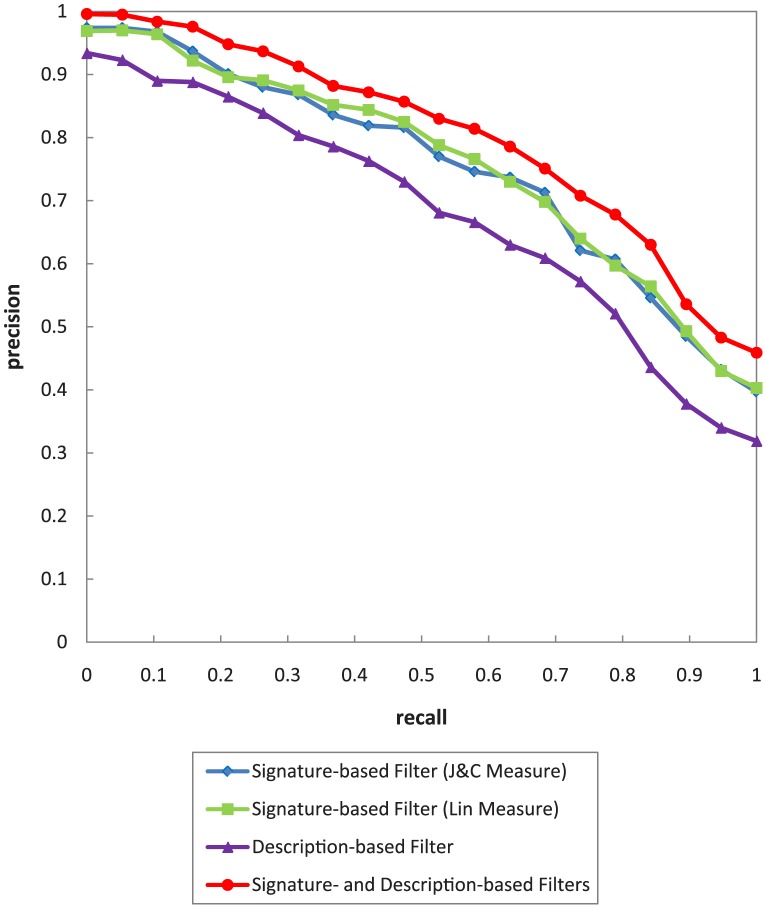
The R/P graph for different variants of UltiMatch-NL.

This experiment evaluates the performance of UltiMatch-NL containing both the Signature-based and Description-based filters. Based on the results in Figure 9, the Signature-based filter relying on the Lin measure is used to configure UltiMatch-NL. In addition, based on the results of analysis in Section 4.1, discriminant analysis method is selected to weight the filters of UltiMatch-NL. The R/P graph is depicted in Figure 12. To compare the performance of UltiMatch-NL variants, their MAPs are provided in Table 7. In addition, the R/P curves of the individual filters are added to Figure 12.

**Table 7:** The MAP of different variants of UltiMatch-NL.

UltiMatch-NL Variant	MAP
Signature-based Filter (J&C Measure)	0.732
Signature-based Filter (Lin Measure)	0.737
Description-based Filter	0.665
Signature- and Description-based Filters	0.790

As shown in Figure 12 and Table 7, the UltiMatch-NL variant with the Signature-based filter outperforms the one with the Description-based filter. In addition, the combination of Signature-based and Description-based filters has the best R/P result compared to the results for each individual variant. This result indicates that UltiMatch-NL performs better when more service description elements are considered for matching. However, this trend depends largely on the availability of data for these elements in the service descriptions.

### 4.3. Analyzing the False Results of UltiMatch-NL

Based on the experimental results, in this section, the primary causes for both false positive and false negative results of UltiMatch-NL are studied and categorized.

#### 4.3.1. Incorrect Estimated Threshold

**Figure 13:**
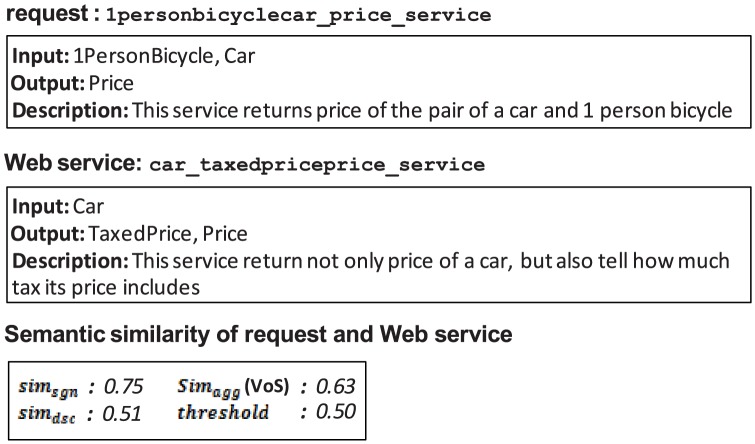
False positive of UltiMatch-NL due to a high estimated threshold.

The similarity threshold value is defined as the measure used to accept or reject the relevance of a Web service to a request. As discussed earlier, this value can be determined manually by a difficult trial-and-error method that is imprecise and may cause incorrect results. An example is shown in Figure 13.

**Figure 14:**
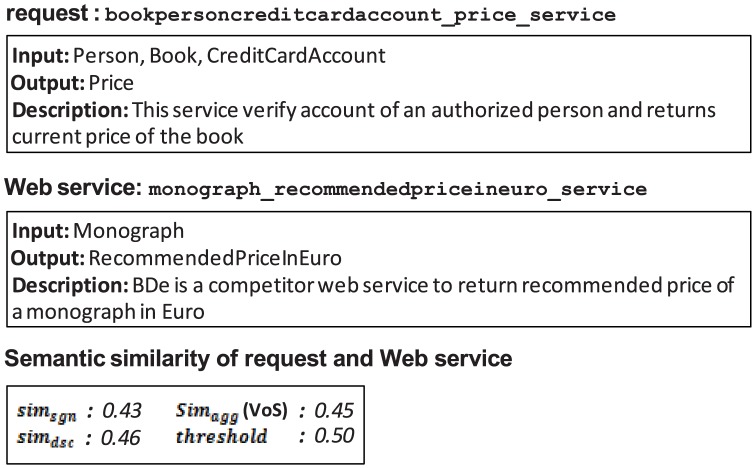
False negative of UltiMatch-NL due to a low estimated threshold.

Both filters of UltiMatch-NL are applied to this request and Web service pairing. The aggregated similarity (in terms of VoS) is calculated as the average of the individual scores. If the determined threshold is less than this value, the Web service is incorrectly considered relevant to the request. To prevent these false positives, the threshold value can be increased. However, this increased threshold may result in false negative answers in the same dataset, as shown in Figure 14.

According to the OWLS-TC, the Web service is relevant to the request in Figure 14. Based on the scores in this figure, their overall similarity is calculated as average. If the similarity threshold is any value greater than this, the Web service is incorrectly considered a false negative result for the request.

As described in earlier, discriminant analysis is adopted in the current experiments to weight the filters of UltiMatch-NL. This method eliminates the need for a manual determination of the similarity threshold. In this case, each value will be automatically estimated with a statistical method and implicitly used to classify the Web services into groups.

#### 4.3.2. Incorrect Calculated Similarity

**Figure 15:**
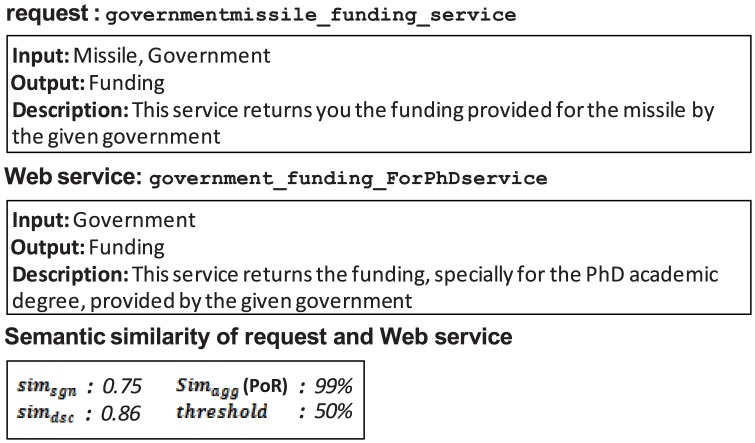
False positive of UltiMatch-NL due to an overestimated similarity.

In some cases, a large variation occurs between the calculated and actual similarities of a request and Web service pairing, forcing UltiMatch-NL to return false results. For example, the request and Web service in Figure 15 are incorrectly considered relevant because they represent a high value of aggregated similarity.

In this case, the calculated similarity between textual descriptions of the request and Web service is overestimated by the Description-based filter due to a shortcoming in the semantic similarity technique adopted by this filter.

Incorrect calculated similarity value is sometimes not because of the applied technique, but may be due to the lack of information or knowledge that it is adopting. In this regard, both of the Non-logic-based filters rely on WordNet to infer the semantic relatedness between a pair of words and to calculate their similarity. Although WordNet is the most complete lexical ontology available, it has the following shortcomings:

- *Undefined compound terms*: Some compound terms such as credit card number, DVD player, full-time, part-time, user name, etc. are missing in WordNet.
- *Undefined abbreviations*: Many abbreviated terms such as ADSL (Asymmetric Digital Subscriber Line), DIVX (DIgital Video eXpress), URI (Uniform Resource Identifier), etc. are not considered in WordNet

In addition, WordNet is unable to dissolve complex terms which are particularly common in programming. Examples are *HospitalPhysician*, *Hiking-place*, and *taxfreeprice*.

**Figure 16:**
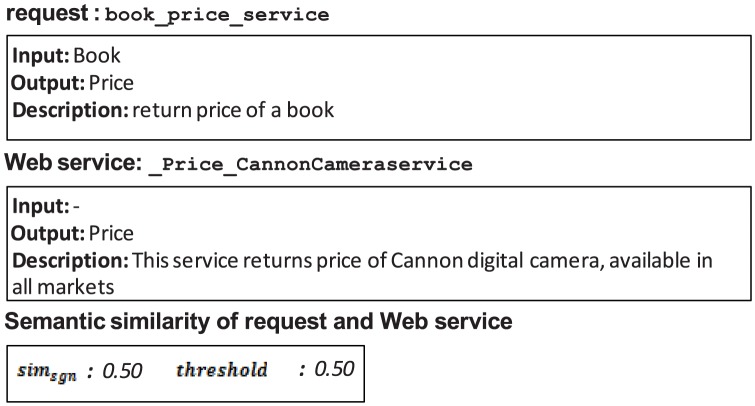
False positive of the Signature-based filter.

Apart from the aforementioned reasons that degrade the performance of the integrated variant of UltiMatch-NL, each individual Signature-based or Description-based variant will return false results if its estimated similarity is incorrect. However, for both variants, some of the false results of each matchmaker are avoided by the other. Thus, applying both of the Non-logic-based filters and properly aggregating their similarity values can improve the performance of the individual variants. An example is depicted in Figure 16.

According to the Signature-based filter, the only matched concept *Price* is sufficient to consider the Web service relevant to the request. This false positive result will be avoided by the Description-based filter because the corresponding textual descriptions are sufficiently different.

### 4.4. Comparative Evaluation of OWLS-MX and UltiMatch-NL

OWLS-MX [50] is a matchmaker for the services described in OWL-S. It uses both logical reasoning and non-logical techniques to search for suitable services. Matching is performed on the input and output parameters of a service profile. OWLS-MX applies several degrees of match (DoMs), namely, *Exact*, *Plug-in*, *Subsumes*, *Subsumed-by*, and *Nearest-neighbor*. Whereas the first three DoMs are purely logic-based, the last two are also applied by the Hybrid matchmaker.

Five variants have been implemented for OWLS-MX. OWLS-M0 only performs Logic-based matching, whereas OWLS-M1 through OWLS-M4 use different similarity metrics for service I/O matching in addition to the Logic-based filter. For the hybrid variants to return a service, a similarity threshold must also be met. The metrics used in these variants are symmetric token-based string similarity measures, such as *cosine*, *extended Jaccard*, *loss of information*, and *Jensen-Shannon*, from the IR.

OWLS-MX2 [11] is the next version of OWLS-MX that integrates non-logic similarity-based matching with all of the logical DoMs except *Exact*. This integration has improved the performance of OWLS-MX by avoiding some cases of Logic-based false positives and negatives. According to [11], the hybrid OWLS-MX2 avoids false positives of non-logical matching. However, as long as the *Nearest-neighbor* exists, this statement is incorrect because false positives of this DoM cannot be avoided by any of the Logic-based filters. However, the logical DoM *Exact* may compensate some of the false negatives of the Non-logic-based matchmaker. OWLS-MX3 [18] is the latest version of OWLS-MX. It is an adaptive matchmaker that learns how to combine various filters for a hybrid semantic selection of services. Nevertheless, the experimental results in [18] demonstrate that OWLS-MX3 is competitive with its predecessor OWLS-MX2 and that it could not significantly improve the previous matchmaker's precision and recall.

In this experiment, UltiMatch-NL is compared to OWLS-MX. To compare the performance of two or more matching approaches, they must be applied to the same set of data. OWLS-MX has been extensively tested over the well-known test collection OWLS-TC. In addition, according to [11] and [18], OWLS-MX2 outperforms other versions of the original matchmaker over the aforementioned test collection. Thus, OWLS-MX2, along with OWLS-TC 2.2 revision 2, is considered for evaluation in this work. For the best configuration of OWLS-MX2, *cosine* is selected as the string similarity measure, *Nearest-neighbor* as the minimum DoM, and a value of 0.7 as the syntactic similarity threshold. These settings are suggested in [11].

**Figure 17:**
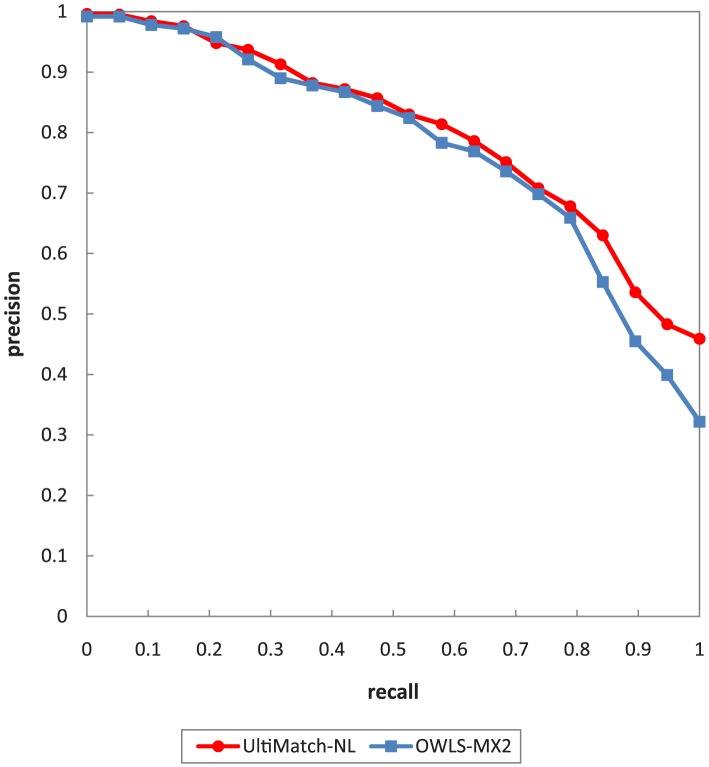
The R/P graph of OWLS-MX2 and UltiMatch-NL.

As the evaluation settings, the standard precision, recall, and MAP are selected. In addition, to be compatible with evaluation results of UltiMatch-NL, the strategy of macro-averaging the individual precision values with 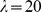 recall steps is adopted. The overall R/P graph is shown in Figure 17. To compare the performance of OWLS-MX2 and UltiMatch-NL, the R/P curve of the latter is added to Figure 17.

Figure 17 demonstrates that UltiMatch-NL outperforms OWLS-MX2 (MAP 0.790 versus 0.763). This result is primarily due to the improved non-logical matching, which avoids some of the false results of OWLS-MX2. The non-logical matching of OWLS-MX2 relies on a single syntactic technique to calculate the similarity of a pair of service descriptions. In contrast, UltiMatch-NL exploits two filters that rely on semantic techniques and aggregates them to predict the relevancy between the Web service and request. In the following, the reasons for failure of OWLS-MX2 are identified and described with typical examples.

#### 4.4.1. False Positives of Logic-based Exact Matching

**Figure 18:**
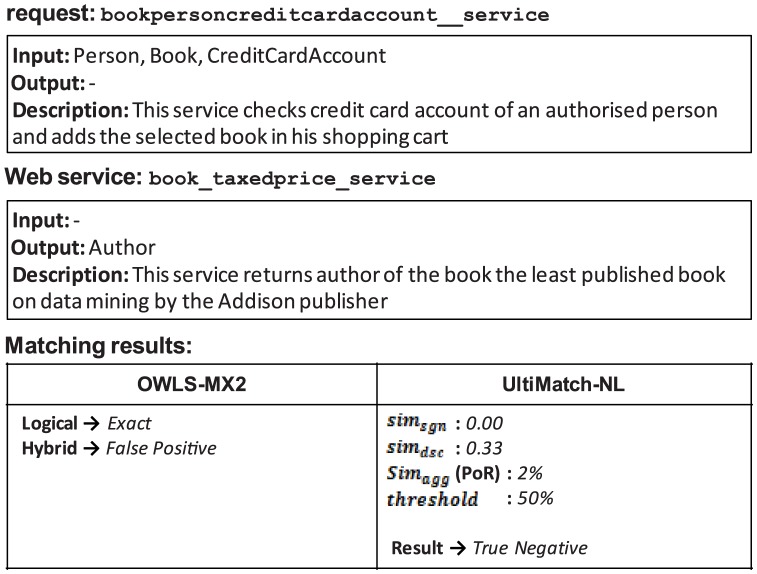
False positive of the logical *Exact* of OWLS-MX2.

Any false positive result of the logical DoM Exact leads in the failure of OWLS-MX2. Because this DoM is not integrated with similarity-based matching, these results could not be eliminated from the answer set of the Hybrid matchmaker. Conversely, UltiMatch-NL avoids some of these false positives. An example is shown in Figure 18.

In this example, UltiMatch-NL eliminates this incorrect result. The aggregated similarity in terms of PoR is considerably less than the threshold.

#### 4.4.2. False Results of Syntactic Similarity-based Matching

**Figure 19:**
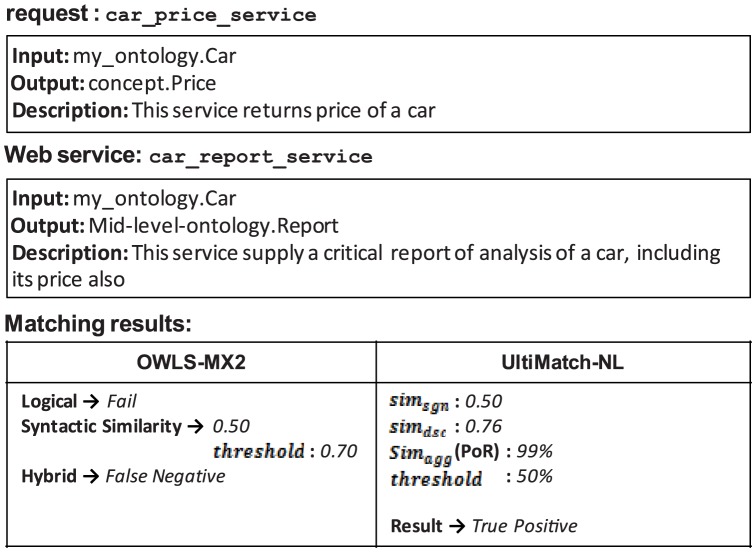
False negative of OWLS-MX2 due to the failure of similarity-based matching to compensate logical *Fail*.

**Figure 20:**
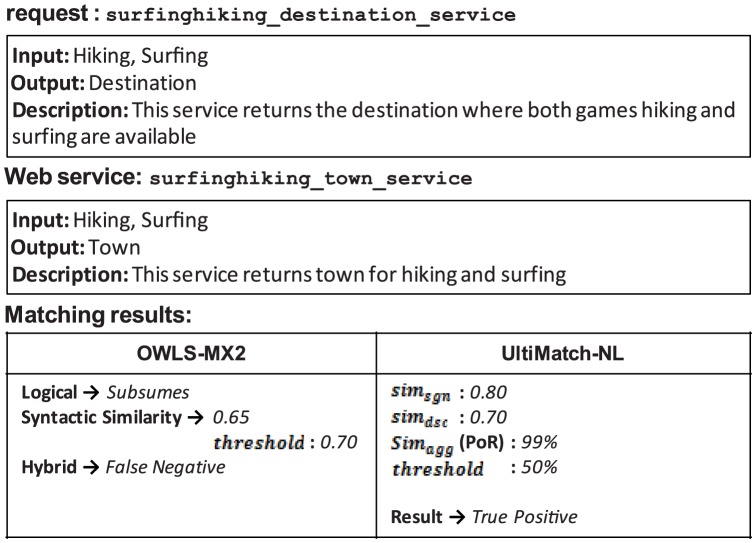
False negative of OWLS-MX2 due to the failure of similarity-based matching to complete the logical DoM.

**Figure 21:**
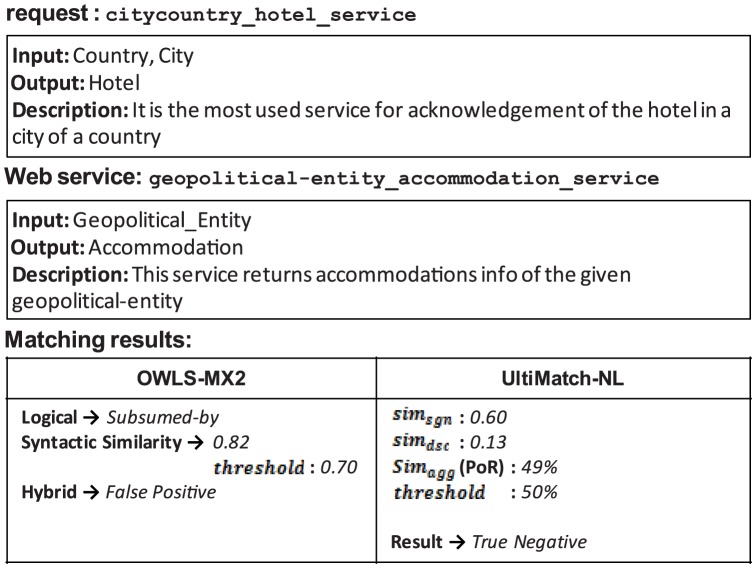
False positive of OWLS-MX2 due to the inability of similarity-based matching to eliminate the logical DoM.

In OWLS-MX2, similarity-based matching is applied to the relaxed DoMs and failure results of the Logic-based matchmaker to complement or compensate these results, respectively. However, any failure to do so would lead to incorrect results from the Hybrid matchmaker. The examples depicted in Figure 19–21 demonstrate different cases that cause the aforementioned failures.

Figure 19 demonstrates that the syntactic similarity between the request and Web service calculated by OWLS-MX2 is insufficient to compensate the incorrect logical *Fail*. In contrast, UltiMatch-NL avoids this false negative result, because it adopts additional filters and predicts the Web service as relevant to the request with a high PoR.

In the case of Figure 20, the logical matching of OWLS-MX2 detects a true relation between the request and Web service, but this result is discarded by the similarity-based matchmaker because the calculated syntactic similarity is insufficient with respect to the configured threshold. However, UltiMatch-NL estimates a true PoR between the mentioned services.

In the case of Figure 21, the logical matching of OWLS-MX2 incorrectly reports the Web service as relevant to the request. This result is not eliminated by the additive syntactic similarity-based matching because the calculated similarity between these services exceeds the threshold. Again, UltiMatch-NL detects a true PoR between the request and Web service.

Apart from the aforementioned cases, UltiMatch-NL outperforms OWLS-MX2 with respect to the following aspects:

- The syntactic similarity threshold in OWLS-MX2 must be set manually. The disadvantages of this requirement are described in Section 3.3. For UltiMatch-NL, this value is determined automatically by the learned classifier method (i.e., the discriminant analysis in the current experiments) based on the known pairs of requests and Web services.
- The syntactic similarity measure in OWLS-MX must be selected by the user. A non-expert user does not know how to choose the most appropriate symmetric token-based string similarity measure.
- To compute the similarity of a pair of service signatures, OWLS-MX2 generates a vector of unfolded input/output concepts to which the syntactic measure is applied. UltiMatch-NL relies on bipartite graph matching to calculate the similarity between two concept sets. In contrast to OWLS-MX2, in this approach, it is possible to determine distinct values for the similarity of input and output concepts, as well as the corresponding concepts of each request and Web service.
- Whereas OWLS-MX2 is only capable of calculating the similarity of concepts that refer to the same ontology, UltiMatch-NL also computes the similarity between concepts of different ontologies.

## Conclusions and Future Work

In this paper, a Non-logic-based semantic matchmaker called UltiMatch-NL is proposed that includes two enhanced filters. These filters namely Signature-based and Description-based consider different elements of the service descriptions for matchmaking. In addition, an automatic weighting method is proposed to aggregate the results of each individual filter and to determine an overall similarity between requests and Web services. In order to predict the relevance of requests and Web services, two classification methods, namely: logistic regression and discriminant analysis are applied and compared. Using this mechanism also eliminates the need for setting a threshold value of similarity and assists the requester in selection among the discovered Web services. In contrast to the majority of the current non-logical approaches that are based on VSM, Ultimatch-NL applies metrics from the field of semantic relatedness to avoid false results. To achieve results of higher accuracy, Ultimatch-NL adapts two of the top-performing IC-based approaches to measure the semantic similarity between concepts in an ontology. In addition, unlike most of the current matchmakers that assume all matching concepts are from the same ontology, Ultimatch-NL allows these concepts to refer to different ontologies. In this case, the similarity of concepts is calculated according to the given terminological relationships of the WordNet ontology.

UltiMatch-NL is experimentally evaluated using the well-known repository of OWLS-TC test collection. For this evaluation, the performance of each of the proposed filters is investigated separately and in combination. The results of each evaluation are encouraging; however the combination of the proposed filters has the best performance compared to the results for each individual variant. In addition, the false positives or negatives that degrade the performance of UltiMatch-NL are thoroughly analyzed and categorized. Moreover, a comparison of UltiMatch-NL and the prominent matchmaker OWLS-MX is presented. The comparative evaluation has shown the superiority of UltiMatch-NL in terms of performance measures.

Future works can incorporate quality of service (QoS) aspects (e.g., availability, reliability, security) in the process of matchmaking. Another extension point could be adapting more mature semantic similarity metrics and advanced natural language processing (NLP) techniques in the proposed filters.
