# Correction: UltiMatch-NL: A Web Service Matchmaker Based on Multiple Semantic Filters

**DOI:** 10.1371/journal.pone.0118386

**Published:** 2015-02-17

**Authors:** The PLOS ONE Staff

There is an error in Fig. 2, “Matching components in a weighted bipartite graph.” Please see the corrected Fig. 2 here.

**Fig 2:**
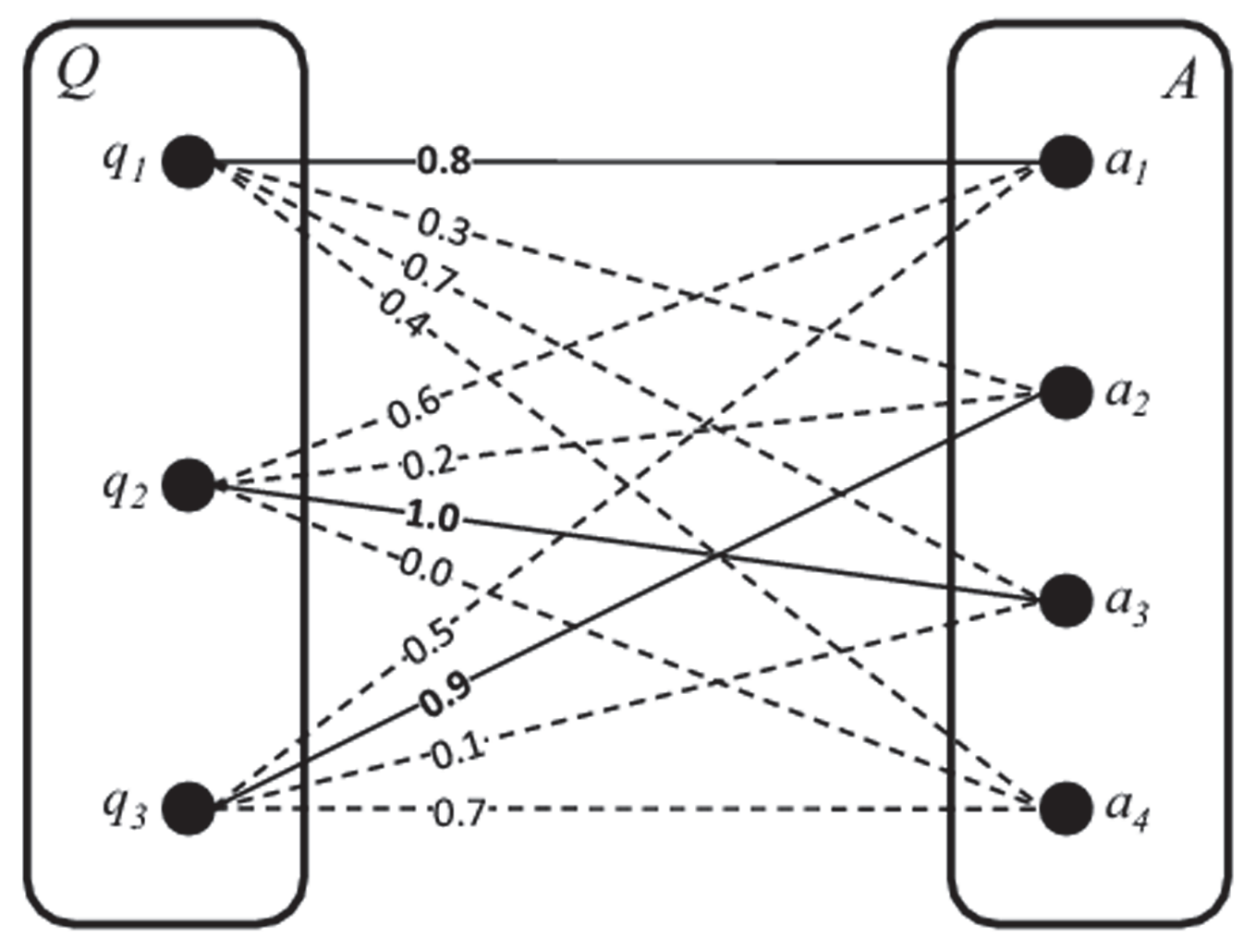
Matching components in a weighted bipartite graph.
